# Efficacy of Combined Desensitization Therapy Based on Protein A Immunoadsorption on Anti-human Leukocyte Antigen Antibodies in Sensitized Kidney Transplant Recipients: A Retrospective Study

**DOI:** 10.7759/cureus.28661

**Published:** 2022-09-01

**Authors:** XiaoFei Chen, YuXian Wang, PeiJian Dong, JiaMei Wang, XiaoTian Yu, BoGuang Yu

**Affiliations:** 1 Nephrology, Shulan (Hangzhou) Hospital, Zhejiang Shuren University Shulan International Medical College, HangZhou, CHN; 2 Research and Development, GuangDong Provincial Key Laboratory of Hemoadsorption Technology, GuangZhou, CHN

**Keywords:** protein a immunoadsorption, desensitization therapy, graft rejection, anti-human leukocyte antigen antibodies, kidney transplantation

## Abstract

Background and objectives

Protein A immunoadsorption (PA-IA) therapy is an immunoglobulin selective apheresis for pre-transplantation desensitization therapy and treatment of post-transplantation antibody-mediated rejection. There is no unified protocol for the timing of PA-IA therapy or its combination with other drug therapy. This study aimed to investigate and analyze the clearance effects of desensitization therapy on human leukocyte antigen (HLA) antibodies to provide a reference for the formulation of clinical desensitization therapy regimens.

Materials and methods

Overall, 27 kidney transplant recipients who received preoperative/postoperative desensitization therapy based on PA-IA therapy in combination with drug therapy were enrolled. The pre-treatment mean fluorescence intensity (MFI) of 1324 human leukocyte antigen (HLA) antibody specificities (MFI >2000) and the post-treatment MFI of the corresponding antibody specificities (after one, four, seven, and 10 sessions) were recorded to analyze the changes in antibody level reduction for the different antibody classes and MFI ranges.

Results

After 10 sessions of PA-IA therapy, the MFI of class I antibodies decreased from 8298.56 to 3196.15 (reduction of 66.80%), while the MFI of class II antibodies decreased from 13,521.09 to 2773.29 (reduction of 71.14%). The pre-treatment level of class II antibodies was significantly higher than that of class I antibodies (p<0.001), whereas the post-treatment levels of class I and II antibodies were comparable (p>0.05). The clearance effects of PA-IA therapy were greater for strongly positive (MFI>10,000) class II antibodies than for strongly positive class I antibodies, showing a reduction of 62.59% (25.17% to 91.04%) and 45.13% (32.70% to 73.94%), respectively (p=0.015).

Conclusions

We confirmed the removal efficacy of PA-IA for HLA antibodies. The removal efficacy of class II antibodies on PA-IA is not inferior to that of class I. Under an adequate number of treatment sessions, the clearance effect of PA-IA therapy for strongly positive class II antibodies may be greater than that for strongly positive class I antibodies.

## Introduction

Anti-human leukocyte antigen (HLA) antibodies are immunoglobulins that target HLAs. In the field of organ transplantation, both preformed antibodies and de novo antibodies can significantly affect the recovery of graft function [1-6]. Moreover, acute and chronic rejections are associated with HLA antibodies, particularly donor-specific antibodies (DSA). Previous studies have suggested that early rejection is significantly more likely to occur in transplant recipients with preoperative preformed HLA antibodies, which is one of the major causes of early graft failure [2,7]. For patients with elevated HLA antibody levels during the perioperative period of kidney transplantation, it is crucial to reduce antibody levels to improve the survival of the kidney allograft. Protein A immunoadsorption (PA-IA) therapy is based on the principle that staphylococcal protein A can specifically bind to human immunoglobulins, thereby removing circulating HLA antibodies and diminishing the damage inflicted on allografts [8-10]. Thus, research on this technique has been expanding in the fields of organ transplantation and autoimmune diseases [11-12]. In kidney transplantation, a previous study [13] revealed that PA-IA therapy can effectively reduce the mean fluorescence intensity (MFI) of HLA antibodies and that it is superior to plasma exchange in reducing HLA antibody levels [14-15]. Researchers have investigated the impact of HLA antibody characteristics (e.g., differences in antibody type, antibody specificity, and titer/MFI) on antibody clearance effects to predict the effectiveness of desensitization therapy and formulate more effective treatment regimens based on the different pre-desensitization antibody characteristics. However, the conclusions of these previous studies are subject to limitations due to their small HLA antibody-specific sample size, an insufficient number of groups, or the inadequate number of treatments. Therefore, the objectives of this study were to investigate and analyze the clearance effects of desensitization therapy on HLA antibodies to provide a reference for the formulation of clinical desensitization therapy regimens. 

## Materials and methods

Patients

A total of 27 patients awaiting kidney transplantation who had received PA-IA therapy in combination with drug therapy at Shulan (Hangzhou) Hospital between December 2019 and December 2021 were enrolled in this retrospective study. This study was conducted in accordance with the Declaration of Helsinki, and approved by the Ethics Committee of Shulan (Hangzhou) Hospital (approval no. KY2022037).

Protein A immunoadsorption therapy

Preoperative PA-IA Therapy

According to the treatment recommendations of the Guidelines on the Use of Therapeutic Apheresis in Clinical Practice from the Writing Committee of the American Society for Apheresis [16], PA-IA therapy was applied during the operation period. Patients who received preoperative treatment (n=16) underwent PA-IA therapy of four to 31 sessions/person (11.5 sessions on average/person) for one to two months before surgery; the treatment frequency was once every other day, and KONPIA® PA-IA columns (KCIA08, Guangzhou Koncen Bioscience Co. Ltd., Guangzhou, China) were employed. After extracorporeal circulation was established, low molecular weight heparin calcium was used for continuous anticoagulation. The extracorporeal blood was first passed through a plasma separator at a blood flow of 100 to 150 mL/min to separate plasma. Thereafter, the separated plasma was loaded onto a PA-IA column at a flow rate of 30 to 40 mL/min via a plasma pump for 15 to 20 minutes of adsorption. Upon completion of adsorption, the bypass was opened, and saline was introduced for plasma re-transfusion at a flow rate of 70 mL/min. Once 350 mL of saline was perfused into the plasma tube, the adsorbed antibodies were eluted using an eluent at a flow rate of 70 mL/min, and the elution ended when the pH ranged from 2.2 to 2.8. Then, the pH was neutralized to 7.0 using a balanced solution, which contained sodium chloride, potassium chloride, sodium citrate, disodium hydrogen phosphate, sodium dihydrogen phosphate (pH range: 6.8 to 7.6), and pre-rinsing was performed again using normal saline to regenerate the adsorption column. After regeneration, the plasma could be loaded onto the column again for further adsorption. One session of treatment included five to 10 continuous cycles of adsorption. The adsorption time was adjusted in accordance with the plasma pump speed, and 500 to 600 mL of plasma was processed in each cycle. Each session of treatment lasted for four to six hours, and the total amount of processed plasma was 3000 to 6000 mL (1.5 to 3 plasma volume).

Postoperative PA-IA Therapy

Patients who received postoperative treatment (n=11) underwent PA-IA therapy four to 18 sessions/person (average, 11.7 sessions/person) for two to 25 days after surgery, using the same treatment regimen as the preoperative treatment group. The treatment sessions were determined according to the patient’s post-treatment (every three sessions) degree of decline in the HLA antibody level. When the decrease in HLA antibody level was higher than 50%, one to three treatment sessions were added, and when it was lower than 50%, three to five immunosorbent treatment sessions were added.

Detection Method of HLA Antibodies in Peripheral Blood

The single-antigen beads assays and Luminex technique were employed for the detection of HLA antibodies. The HLA antibody specificities with an MFI of >500 were identified as positive, and antibodies with an MFI of 500 to 2000 were completely eliminated after treatment. Therefore, only those with an MFI of >2000 were selected for investigation in this study, and 1324 eligible data points were finally selected. The primary endpoint was the reduction in antibody MFI (initial MFI to MFI after treatment/initial MFI), while post-clearance negative conversion (MFI <500) was defined as a reduced rate of 100%. The secondary endpoint was the reduction in post-treatment MFI to <2000, which indicated that the treatment was effective. Changes in the MFI at different HLA antibody specificities were measured before treatment and after the first, fourth, seventh, and 10th PA-IA sessions.

Antibody Induction Therapy Regimen

Pre-transplantation desensitization therapy was performed once using rituximab (MabThera, dose: 100 mg/time, timing: after completion of the first immunoadsorption therapy). Rituximab (MabThera) was administered once (100 mg/time) intraoperatively. After transplantation, rituximab (dose: 100 mg/time) was administered intermittently once or twice in the early phase of treatment (within one week after surgery) depending on the postoperative HLA antibody titers. Patients whose preoperative HLA antibody titers were positive (MFI 2000 to 10000) and strongly positive (MFI >10,000) received 100 mg and 200 mg of rituximab after transplantation, respectively, although this was administered a maximum of four times in total during and after transplantation.

Intravenous immunoglobulin was administered once before surgery (dose: 20 g/time), and once a day after surgery (dose: 10 g/time) for five consecutive days. The dosage for patients who had a high body weight was adjusted according to the increase in body weight. Rabbit anti-human thymocyte globulin (ATG) was administered four times perioperatively, including 50 mg preoperative induction administered two to four hours before transplantation. After transplantation (early phase), rabbit ATG was administered once every other day (25 mg/time) three times.

Statistical Analyses

Analyses were performed using the median (first/third quartiles) or mean (standard deviation) as appropriate. Measurement data were subjected to the Kolmogorov-Smirnov normality test and Levene’s test to examine the homogeneity of variance. Parametric tests were performed on data that satisfied the assumptions of normal distribution and homogeneity of variance. Non-parametric tests were performed on data that did not satisfy these assumptions, which were described using the median (first quartile-third quartile) and subjected to the Kruskal-Wallis H test for the comparison of between-group differences. A comparison of antibody levels at different time points was performed using the generalized estimating equation method. Statistical analyses were performed with R language environment version 4.0.3 (The R Foundation, Indianapolis, IN, USA).

## Results

Patient characteristics

We included a total of 27 transplant recipients: nine male and 18 female patients aged 26 to 63 years (mean age, 46.3 years). Among them, 10 patients had a history of kidney transplantation, eight patients had a history of blood transfusion, 16 female patients had a history of pregnancy, and the remaining patients had other causes of sensitization (e.g., long-term dialysis). Kidney recipients enrolled in this study were excluded from autoimmune disease or immune deficiency. The ABO blood types of the donors and recipients were compatible, and only cadaveric donor kidneys were provided. The warm ischemia time was 4.9±1.6 min, whereas the cold ischemia time was 10.2±4.0 h.

Clearance effects for HLA class I and HLA class II antibody specificities

A total of 1324 data points for HLA class I and HLA class II antibody specificities (initial MFI >2000) were analyzed in this study: class I (n=724) and class II (n=600). Table 1 shows the distribution of the median antibody levels (MFI) and reduced rates of the two classes of antibodies, wherein data are presented as medians (first-third quartiles). After one to 10 sessions of immunoadsorption (IA) therapy, 582 antibody specificities yielded successful treatment (post-treatment MFI <2000), accounting for 44% of the total. Among them, the pre-treatment median MFI of class I antibody (n=334, 46%) and class II antibody (n=248, 41%) was 5304.32 (3436.81 to 8408.53) and 6773.70 (3378.20 to 14,487.86), respectively. More HLA antibodies were cleared for the weakly positive class II group (n=76, 88.37%) than for the weakly positive class I group (n=117, 77.48%). There were no significant differences in the clearance effects between class I and II antibodies for the moderately and strongly positive groups (p>0.05) (Table 2).

**Table 1:** Antibodies yielding successful treatment (post-treatment MFI <2000) * median (first-third quartiles) Post-1: Median MFI of antibodies after the first IA therapy; Post-4, Post-t-7, and Post-t-10: Represent the median MFI of antibodies after the fourth, seventh, and 10th sessions of IA therapy, respectively. Reduction-1, Reduction-4, Reduction 7, and Reduction-10: Represent the reduction in HLA class I and HLA class II after the first, fourth, seventh, and 10th sessions of IA therapy, respectively. IA: Immunoadsorption, MFI: Mean fluorescence intensity, HLA: Human leukocyte antigen

	Class I (n=334, 46%)		Class II (n=248, 41%)	
Initial MFI*	5304.32 (3436.81–8408.53)		6773.70 (3378.20–14,487.86)	
Post-1 MFI*	2423.75 (1469.99–5161.84)		3004.00 (1384.35–12,429.43)	
Post-4 MFI*	987.01 (391.41–1832.35)		1243.47 (546.87–6159.25)	
Post-7 MFI*	645.91 (226.90–1274.76)		799.41 (339.84–2412.55)	
Post-10 MFI*	457.35 (201.17–1,071.41)		257.76 (0.00–918.77)	
Reduction-1	46.08% (12.91%–69.97%)		47.79% (22.69%–69.49%)	
Reduction-4	82.66% (68.67%–93.18%)		77.92% (48.83%–89.15%)	
Reduction-7	89.52% (77.01%–96.41%)		87.19% (74.97%–93.63%)	
Reduction-10	91.01% (84.59%–95.37%)		96.50% (83.70%–100.00%)	
Initial MFI*	n (%)	n (%)		p-value
2000–4000	117 (77.48)	76 (88.37)		<0.05
4000–10,000	152 (56.72)	81 (57.45)		>0.05
>10,000	65 (21.24)	91 (24.40)		>0.05

**Table 2:** Comparison and clearance effect for HLA class I and HLA class II antibodies * median (first-third quartiles) Post-1: Median MFI of antibodies after the first IA therapy; Post-4, Post-t-7, and Post-t-10: Represent the median MFI of antibodies after the fourth, seventh, and 10th sessions of IA therapy, respectively. Reduction-1, Reduction-4, Reduction 7, and Reduction-10: Represent the reduction in HLA class I and HLA class II after the first, fourth, seventh, and 10th sessions of IA therapy, respectively. IA: Immunoadsorption, MFI: Mean fluorescence intensity, HLA: Human leukocyte antigen

	Class I	Class II	p-value
Initial MFI*	8298.56 (4450.38–14,003.05)	13,521.09 (6153.58–20,440.01)	<0.001
Post-1 MFI*	5425.02 (2260.81–11,624.31)	10,567.54 (2660.24–16,851.67)	<0.001
Post-4 MFI*	3310.28 (995.53–7400.64)	9278.33 (1243.47–16,434.13)	<0.001
Post-7 MFI*	1965.22 (645.91–7862.10)	4349.39 (802.01–13,260.19)	<0.001
Post-10 MFI*	3200.68 (674.90–9281.40)	2773.29 (415.95–11,192.52)	0.617
Reduction-1	41.55% (7.13%–58.77%)	26.46% (3.20%–53.19%)	0.031
Reduction-4	62.37% (25.00%–86.78%)	43.42% (5.31%–78.85%)	<0.001
Reduction-7	66.63% (41.05%–94.53%)	62.76% (19.17%–88.53%)	0.014
Reduction-10	66.80% (33.17%–90.48%)	71.14% (26.95%–100.00%)	0.371
	p<0.001	p<0.001	

**Figure 1:**
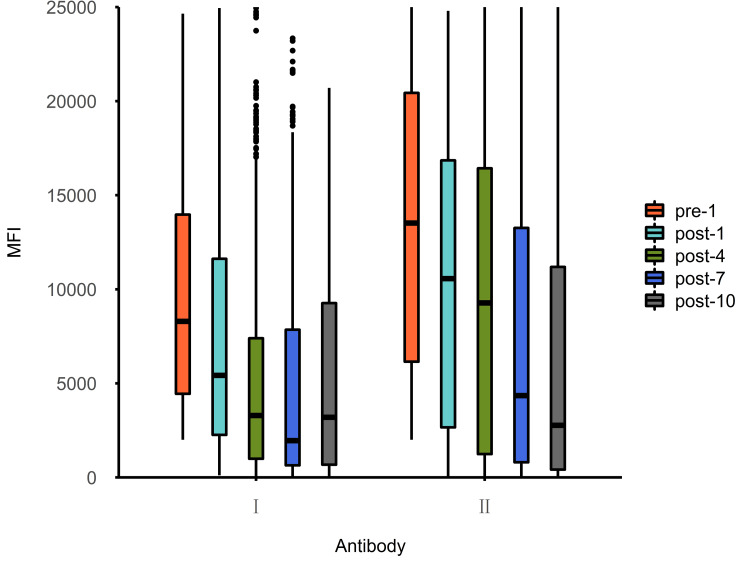
Median MFI values of HLA class I and HLA class II antibody specificities pre-1: Initial MFI of antibodies; post-1: Median MFI of antibodies after first IA therapy; post-4, post-7, and post-10: Represent the median MFI of antibodies after the fourth, seventh, and 10th IA therapy, respectively. HLA: Human leukocyte antigen, IA: Immunoadsorption. MFI: Mean fluorescence intensity

**Figure 2:**
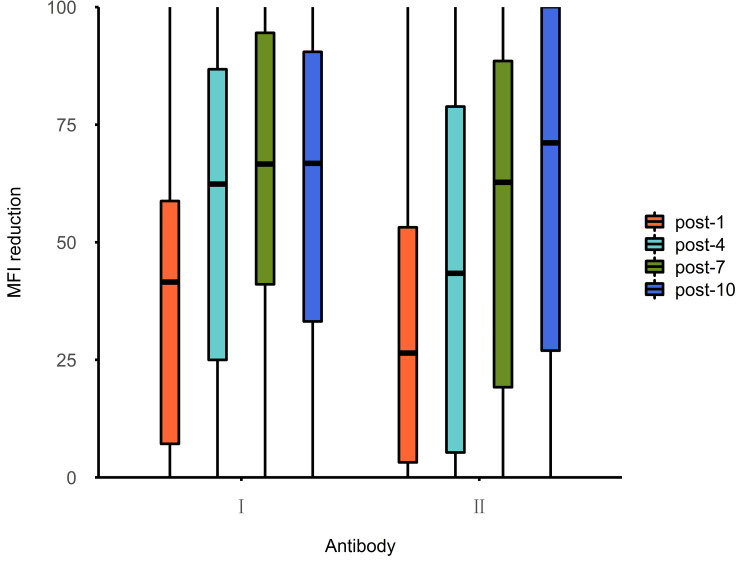
Reduction rates of HLA class I and HLA class II antibody specificities after treatment post-1: Represents the reduction rate after the first IA therapy; post-4, post-7, and post-10: Represent the reduction rates after the fourth, seventh, and 10th IA therapy, respectively. HLA: Human leukocyte antigen, IA: Immunoadsorption. MFI: Mean fluorescence intensity

Before treatment, the median MFI of class II antibodies was significantly higher than that of class I, and the median MFI of class I and II antibodies was 8298.56 (4450.38 to 14,003.05) and 13,521.09 (6153.58 to 20,440.01), respectively (p<0.001). In the early phase of PA-IA therapy (sessions one to seven), class I antibodies were cleared more quickly, showing a greater reduction than that of class II in the first seven sessions (the most significant difference occurred after four sessions, p<0.001). In the late phase of PA-IA therapy (session 10), the clearance effects for class II antibodies increased gradually as the number of sessions increased, and the difference compared with class I antibodies became insignificant, with class I and II antibodies showing a reduction of 66.80% (33.17% to 90.48%) and 71.14% (26.95% to 100.00%), respectively (p>0.05). Additionally, after the PA-IA therapy (session 10), the levels of class I and II antibodies were essentially the same at 3196.15 (676.54 to 9266.92) and 2773.29 (415.95 to 11,192.52), respectively (p=0.617) as seen above in Table 1 and Figures 1-2 below.

Analysis of the clearance efficiencies for different antibody specificities

For class I antibodies, the differences in the initial MFI value and reduction rates were not significant (p=0.27 and p=0.356), whereas class II antibodies showed varying reduction rates at different loci. More specifically, the clearance effect for the anti-HLA-DP antibodies was relatively high, with a reduction of 96.27% (74.25% to 100%), while the anti-HLA-DR and anti-HLA-DQ antibodies showed relatively high initial antibody values at 14,027.55 (5873.79 to 20,331.43) and 15,380.31 (9596.51 to 21,092.58), respectively (p=0.083), with a reduction of 66.03% (37.33% to 84.23%) and 54.11% (11.12% to 94.17%), respectively (p=0.265) (Table 3). There were no significant differences in the clearance effects of IA therapy for class Ⅱ antibodies against HLA-DR and HLA-DQ molecules.

**Table 3:** Comparison and clearance effect for different antibody specificities * median (first-third quartiles) MFI: Mean fluorescence intensity, HLA-A: Antibody for human histocompatibility antigen-A, HLA-B: Antibody for human histocompatibility antigen-C, HLA-DR: Antibody for human histocompatibility antigen-DR, HLA-DQ: Antibody for human histocompatibility antigen-DQ, HLA-DP: Antibody for human histocompatibility antigen-DP, post-10: Post the 10th session of treatment

	N	n%	Initial MFI*	Median MFI*(post-10)	Reduction (post-10)
Class I	724		8298.56 (4450.38–14,003.05)	3196.15 (676.54–9266.92)	66.81% (33.14%–87.73%)
HLA-A	169	23%	8479.21 (4576.98–13,208.07)	4037.44 (470.15–8191.46)	54.11% (37.51%–88.37%)
HLA-B	506	70%	8333.68 (4457.44–14,520.55)	3184.83 (771.94–11,210.42)	71.49% (28.83%–87.71%)
HLA-C	49	7%	6748.46 (4116.95–11,451.12)	1381.96 (775.25–3293.31)	74.64% (63.70%–85.86%)
p-value			0.27	0.028	0.36
Class II	600		13,521.09 (6153.58–20,440.01)	2773.29 (415.95–11,192.52)	71.14% (26.95%–93.53%)
HLA-DR	258	43%	14,027.55 (5873.79–20,331.43)	3029.33 (1164.87–10,203.64)	66.03% (37.33%–84.23%)
HLA-DQ	214	36%	15,380.31 (9596.51–21,092.58)	5926.27 (555.90–13,428.37)	54.11% (11.12%–94.17%)
HLA-DP	128	21%	9014.01 (5779.66–18,512.58)	226.65 (0.00–3638.98)	96.27% (74.25%–100%)
p-value			0.001	<0.001	<0.001

Antibody clearance effects within different initial MFI subgroups

**Figure 3:**
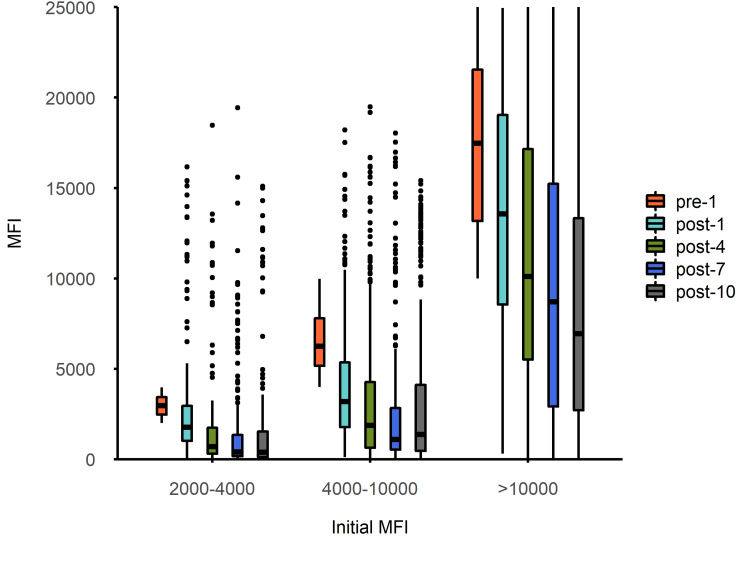
Overall change in median MFI in each positive group pre-1: Initial MFI of antibodies; post-1: Median MFI of antibodies after first IA therapy; post-4, post-7, and post-10: Represent the median MFI of antibodies after the fourth, seventh, and 10th IA therapy, respectively; IA: Immunoadsorption. MFI: Mean fluorescence intensity

**Figure 4:**
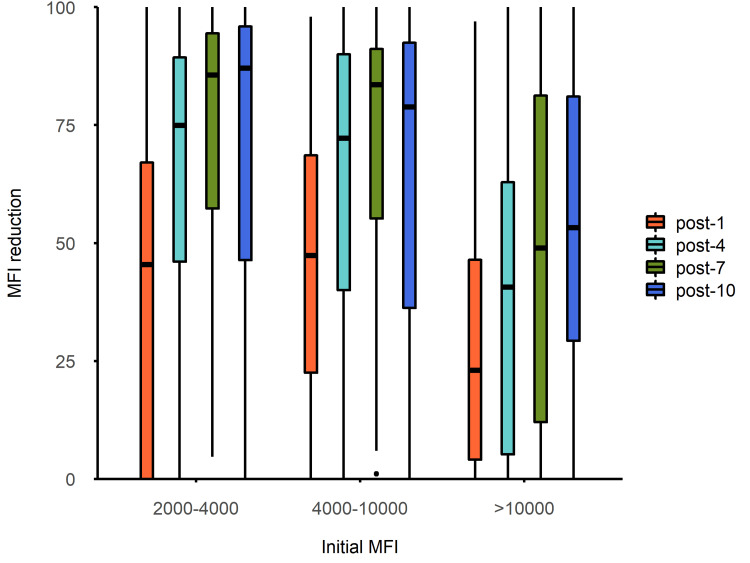
Overall change in reduction rate in each positive group post-1: Represents the reduction rate after the first IA therapy; post-4, post-7, and post-10: Represent the reduction rates after the fourth, seventh, and 10th IA therapy, respectively; IA: Immunoadsorption, MFI: Mean fluorescence intensity

Based on the pre-treatment range of the MFI, we established the weakly positive group (MFI 2000 to 4000), moderately positive group (MFI 4000 to 10,000), and strongly positive group (MFI >10,000). Figures 3-4 show the overall changes (median MFI and reduction rates, respectively) of each positive group. The initial MFI values of class I and II antibodies in the weakly positive group (n=237, 17.5%) and moderately positive group (n=409, 30.5%) were basically comparable (the differences were not statistically significant). In the early phase of the PA-IA therapy (sessions one to seven), the reduction rates for class II antibodies in the weakly positive group and the moderately positive group were higher than those for class I antibodies (with more significant differences in the moderately positive group). However, in the late phase of the PA-IA therapy (session 10), the reduction rates for class I and II antibodies were generally comparable in the weakly positive and moderately positive groups. Weakly positive group: 100% (34.12% to 100%) for class I antibodies vs. 100% (48.09% to 100%) for class II antibodies (p=0.342); moderately positive group: 81.10% (42.89% to 100%) for class I antibodies vs. 73.35% (-21.39% to 100%) for class II antibodies (p=0.358) (Table 4). Therefore, the differences in the clearance rates of the PA-IA therapy for class I and II antibodies in the weakly positive and moderately positive groups were mainly due to the faster clearance of class II antibodies in the early phase of treatment, followed by a more comparable trend between the two in the late phase of treatment.

**Table 4:** Clearance effect for HLA antibodies of each positive group * median (first-third quartiles) MFI: Mean fluorescence intensity, post-10: Post the 10th immunoadsorption (IA) therapy session

	n	n%	Median MFI* (initial)	Median MFI* (post-10)	Reduction (post-10)
Class I	724		8298.56 (4450.38–14,003.05)	3196.15 (676.54–9266.92)	
2000–4000	151	21%	3065.50 (2524.65–3532.24)	356.43 (154.21–2023.83)	100% (34.12%–100%)
4000–10000	268	37%	6206.84 (5135.45–7821.33)	1343.98 (485.88-3876.78)	81.10% (42.89%–100%)
>10000	306	42%	15,891.21 (12,186.45–20,640.62)	7443.74 (3847.68–12,796.36)	45.13% (32.70%–73.94%)
	n	n%	Median MFI (initial)	Median MFI (post-10)	Reduction (post-10)
Class II	600		13,521.09 (6153.58–20,440.01)	2773.29 (415.95–11,192.52)	
2000–4000	86	14%	2884.69 (2301.43–3302.98)	449.41 (26.34–1479.95)	100% (48.09%–100%)
4000–10000	141	24%	6443.40 (5227.05–7764.28)	1524.13 (312.62–7466.54)	73.35% (–21.39%–100%)
>10000	373	62%	18,682.86 (14,421.24–22,156.76)	6472.68 (1474.07–14,309.32)	62.59% (25.17%–91.04%)

In the strongly positive group (n=679, 52%), the initial value of class II antibodies was greater than that of class I antibodies, at 18,682.86 (14,421.24 to 22,156.76) and 15,891.21 (12,186.45 to 20,640.62), respectively (p<0.001). The decreasing trends (changes in reduction) of class I and II antibodies in the strongly positive group were consistent with the overall trend of decline. Specifically, the reduction in class I antibody levels in the strongly positive group was greater in the early phase of IA therapy (sessions one to seven) (p<0.001), whereas the reduction in class II antibody levels in the strongly positive group was more significant at the late phase of IA therapy (10th session), with class II and I antibodies in the strongly positive group showing a reduction of 62.59% (25.17% to 91.04%) and 45.13% (32.70% to 73.94%), respectively (p=0.015) (Table 5).

**Table 5:** Comparison and clearance effect for HLA class I and class II antibodies in the strongly positive group Pre-1: Initial MFI of antibodies, Post-1: Median MFI of antibodies after the first IA therapy; Post-4, Post-t-7, and Post-t-10: Represent the median MFI of antibodies after the fourth, seventh, and 10th sessions of IA therapy, respectively. Reduction-1, Reduction-4, Reduction 7, and Reduction-10: Represent the reduction in HLA class I and HLA class II after the first, fourth, seventh, and 10th sessions of IA therapy, respectively. IA: Immunoadsorption, MFI: Mean fluorescence intensity, HLA: Human leukocyte antigen

MFI >10000 (n=679, 52%)	Class I (n=306)	Class II (n=373)	p-value
Pre-1	15,891.21 (12,186.45–20,640.62)	18,682.86 (14,421.24–22,156.76)	<0.001
Post-1	11,586.15 (6725.16–17,679.81)	15,298.69 (11,060.93–20,170.10)	<0.001
Post-4	6484.88 (4070.16–10,736.73)	12,959.11 (9095.25–20,260.47)	<0.001
Post-7	7280.13 (1193.18–12,059.03)	10,096.32 (4971.14–18,653.92)	<0.001
Post-10	7443.74 (3847.68–12,796.36)	6472.68 (1474.07–14,309.32)	0.057
Reduction-1	39.86% (7.38%–53.24%)	14.11% (1.86%–31.92%)	<0.001
Reduction-4	59.06% (21.07%–69.38%)	21.61% (3.40%–48.08%)	<0.001
Reduction-7	52.92% (28.53%–93.09%)	43.16% (5.83%–68.45%)	<0.001
Reduction-10	45.13% (32.70%–73.94%)	62.59% (25.17%–91.04%)	0.016

Correlation analysis of antibody reduction and initial value

**Figure 5:**
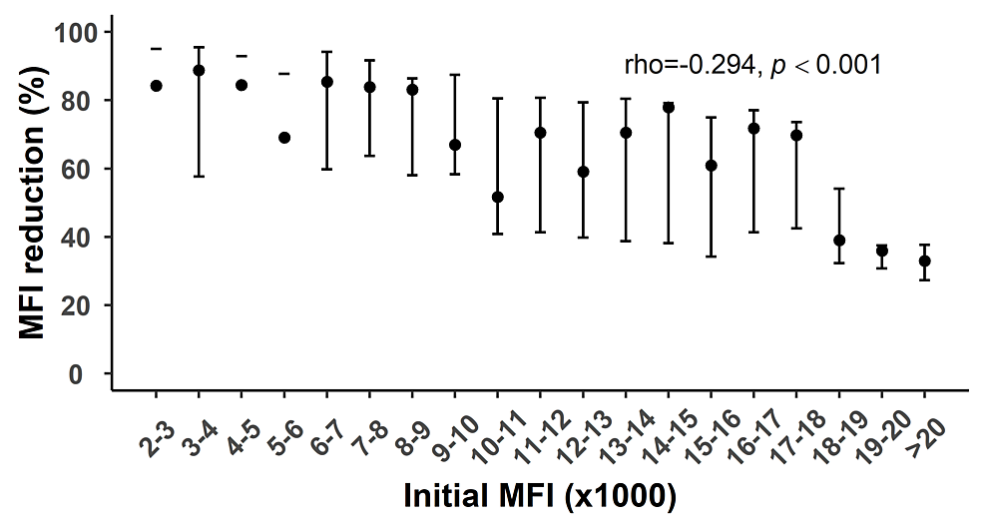
Relationship between initial MFI and MFI reduction in class I after the 10th round of IA therapy MFI: Mean fluorescence intensity, IA: Immunoadsorption

**Figure 6:**
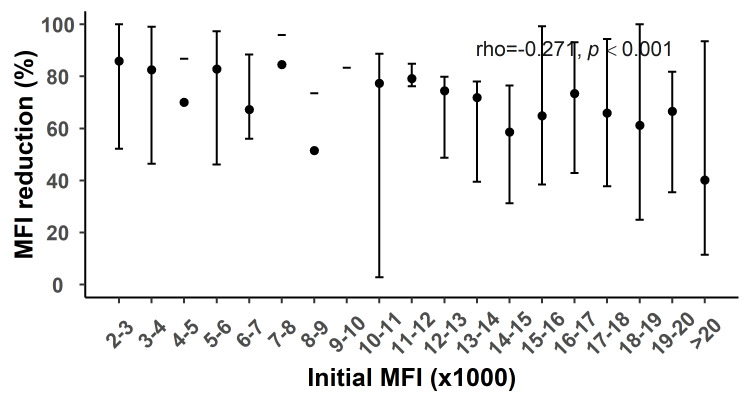
Relationship between initial MFI and MFI reduction in class II after the 10th round of IA therapy MFI: Mean fluorescence intensity, IA: Immunoadsorption

The results of correlation analysis revealed that the reduction in antibody levels corresponding to the different number of treatment sessions was correlated with the initial MFI level; more specifically, antibody reduction was negatively correlated with the initial value (rho<0). Table 6 shows the correlation coefficients for the reduction in different antibody classes and their initial values. Figures 5-6 show the correlation analysis plots for the initial MFI and the reduction in class I and II antibody levels after 10 treatments, respectively. After the seventh and 10th treatment sessions, the degree of negative correlation between the reduction in class I antibody levels and their initial level was stronger as the number of treatment sessions increased. This may be associated with the rapid reduction in class I antibody levels in the early phase of the PA-IA therapy and the decreased reduction in class I antibody levels in the strongly positive group in the late phase of the PA-IA therapy. In contrast, although the reduction in class II antibodies was negatively correlated with the initial level, the degree of negative correlation between them decreased as the number of treatments increased, indicating that the clearance efficiency for strongly positive class II antibodies increased as the number of treatment sessions increased.

**Table 6:** Relationship between initial MFI and MFI reduction in HLA class I and HLA class II after the first, fourth, seventh, and 10th sessions of IA therapy Antibody reduction was negatively correlated with the initial value (rho<0) MFI: Mean fluorescence intensity, HLA: Human leukocyte antigen, IA: Immunoadsorption

Class I		Reduction-1	Reduction-4	Reduction-7	Reduction-10
Initial MFI	rho	-0.013	-0.068	-0.088	-0.294
	p	0.771	0.074	0.028	<0.001
Class II					
Initial MFI	rho	-0.448	-0.537	-0.532	-0.131
	p	0.000	0.000	0.000	0.005

## Discussion

In this retrospective study, we performed a thorough analysis of the differences in antibody clearance among different classes, initial MFI subgroups, and different treatment phases, and our findings confirmed the clearance effects of PA-IA on HLA antibodies. Regarding differences in the clearance effects for class I and II antibodies, our results demonstrated that when an adequate number of treatment sessions were completed, the clearance effects for class I antibodies were greater than those for class II antibodies in the early to middle phases of IA therapy (before seven sessions), whereas no significant differences were found in the clearance effects between the two in the late phase of IA therapy (after 10 sessions). This was most likely because the MFI of class II antibodies was generally higher than that of class I antibodies, and class II antibodies (particularly HLA-DQ antibodies) were more likely to become saturated [16]. Our study results also showed that the initial MFI of class II antibodies (particularly HLA-DQ antibodies) was significantly higher than that of class I antibodies. Therefore, we believe that there is no difference in the clearance effects of PA-IA therapy on class I and II antibodies; however, the generally higher MFI of class II antibodies (particularly DQ antibodies) and the inadequate number of treatment sessions contributed to the lower clearance rate of IA therapy or plasma exchange for class II antibodies in clinical practice [14]. Furthermore, a study employing a titration assay for the determination of HLA antibody levels reported no significant difference in the clearance effects of plasma exchange on class I and II antibodies [16]

Interestingly, our findings showed that the clearance effects for weakly and moderately positive class II antibodies were greater than those for weakly and moderately positive class I antibodies in the early to middle phases of IA therapy, whereas the clearance effects remained consistent between the two classes in the late phase of IA therapy. Additionally, for strongly positive antibodies, the clearance effects for class I antibodies were greater than those for class II antibodies in the early to middle phases of IA therapy, whereas the clearance effects for class II antibodies were greater than those for class I antibodies in the late phase of IA therapy. It is possible that for strongly positive antibodies, the reduction in class II antibodies could not be truly revealed as they were more easily saturated in the early phase of IA therapy, although the clearance effects for class II antibodies became greater than those for class I antibodies once the saturation limit was surpassed. Therefore, the PA-IA therapy has the potential to achieve more effective clearance of class II antibodies than of class I antibodies. Nevertheless, this result awaits further validations and may be related to the more severe rebound of strongly positive class I antibodies. 

Jambon et al. [15] argued that antibody clearance effects are subjected to the initial antibody values rather than the antibody type or specificity, and that antibody clearance efficiency is negatively correlated with the initial value, which is consistent with our study’s conclusions. Moreover, we added the number of treatments to the correlation analysis between the initial value and clearance rate of different types of antibodies and found that the antibody clearance efficiency was subjected to multivariate rather than univariate effects. This conclusion was supported by the results of correlation analysis between the initial values and reduction in antibody levels at different phases of treatment. Specifically, the clearance efficiency for class I antibodies was greater than that for class II antibodies in the strongly positive group in the early phase of the PA-IA therapy, whereas the clearance effects for strongly positive class II antibodies gradually became more prominent with an increasing number of treatment sessions in the late phase of IA therapy treatment, as demonstrated by the reduction in the absolute values of the correlation coefficients and the smaller difference in reduction between class I and II antibodies in the strongly positive group.

Currently, studies on the evaluation of kidney allograft function following desensitization therapy based on PA-IA are still relatively scarce. Böhmig et al. [9] performed perioperative treatment of pre-sensitized cadaveric donation transplant recipients using PA-IA therapy in combination with immunosuppressive therapy including cyclosporin A and rabbit antithymocyte globulin. They reported that five years after transplantation, the death-censored graft survival, overall graft survival, and patient survival were 76%, 63%, and 87% respectively, with no significant differences among the three groups of patients (CDCXM+/DSA+, n=21; CDCXM-/DSA+, n=30; CDCXM-/DSA-, n=17) [17]. A randomized controlled trial [8] reported in 2007 showed that PA-IA therapy was effective at reversing severe C4d-positive antibody-mediated rejection, and the proportion of patients requiring no dialysis in the experimental group was significantly higher than that in the control group 21 days after treatment. With respect to the 24 patients who completed kidney transplantation in this study, their serum creatinine (sCr) levels showed a steady downward trend, while their estimated glomerular filtration rate showed a steady upward trend two years after surgery.

In clinical practice, it was usually observed that class II antibodies were more difficult to abolish than class I, which led to the false conclusion that some therapeutic methods had lower removing efficacy for class II antibodies than for class I. Some studies had revealed that the phenomenon maybe due to the generally higher MFI and more frequent saturation of class II antibodies [18]. However, few studies compared the efficacy of therapy regimens between classes I and II. Our study compared the removal efficacy of PA-IA between classes I and II. We found that the removal efficacy of PA-IA for class II antibodies was not inferior to that for class I. Moreover, a lower or acceptable level could be achieved for even strongly positive class II antibodies wherein adequate adsorption sessions were performed (approximately >10 sessions). These results provide an important reference for physicians employing PA-IA for transplant recipients with positive HLA II antibodies, especially those in the strongly positive group.

Nevertheless, this study has some limitations, including its retrospective nature rather than a prospective one, the non-inclusion of a control group receiving no PA-IA therapy, and an imbalance between the baseline characteristics of class I and II antibodies. Moreover, because of the scarcity of donor kidneys and the difficulty associated with highly sensitized kidney transplantation, only 27 recipients were enrolled in this study. Therefore, further prospective controlled trials and studies with a relatively larger sample size are warranted to validate the findings of our study.

## Conclusions

In this study, we confirmed the removal efficacy of PA-IA for HLA antibodies. The results indicated that the removing efficacy of class II antibodies on PA-IA is not inferior to that of class I. This implies that desensitization therapy based on PA-IA is clinically effective in ensuring the successful completion of kidney transplantation and the stable recovery of postoperative renal allograft function. Future prospective and control trials are required to validate the above conclusions.
